# Recent studies of the effects of microgravity on cancer cells and the development of 3D multicellular cancer spheroids

**DOI:** 10.1093/stcltm/szaf008

**Published:** 2025-03-18

**Authors:** Daniela Grimm, Thomas J Corydon, Jayashree Sahana, Luis Fernando González-Torres, Armin Kraus, Shannon Marchal, Petra M Wise, Ulf Simonsen, Marcus Krüger

**Affiliations:** Department of Microgravity and Translational Regenerative Medicine, Otto-von-Guericke-University, 39106 Magdeburg, Germany; Research Group “Magdeburger Arbeitsgemeinschaft für Forschung unter Raumfahrt-und Schwerelosigkeitsbedingungen” (MARS), Otto-von-Guericke-University, 39106 Magdeburg, Germany; Department of Biomedicine, Aarhus University, 8000 Aarhus C, Denmark; Department of Biomedicine, Aarhus University, 8000 Aarhus C, Denmark; Department of Ophthalmology, Aarhus University Hospital, 8200 Aarhus N, Denmark; Department of Biomedicine, Aarhus University, 8000 Aarhus C, Denmark; Department of Microgravity and Translational Regenerative Medicine, Otto-von-Guericke-University, 39106 Magdeburg, Germany; Clinic for Plastic, Aesthetic and Hand Surgery, Otto-von-Guericke-University, 39120 Magdeburg, Germany; Department of Microgravity and Translational Regenerative Medicine, Otto-von-Guericke-University, 39106 Magdeburg, Germany; Department of Microgravity and Translational Regenerative Medicine, Otto-von-Guericke-University, 39106 Magdeburg, Germany; Research Group “Magdeburger Arbeitsgemeinschaft für Forschung unter Raumfahrt-und Schwerelosigkeitsbedingungen” (MARS), Otto-von-Guericke-University, 39106 Magdeburg, Germany; The Saban Research Institute, Children’s Hospital Los Angeles, University of Southern California, Los Angeles, CA 90027, United States; Department of Biomedicine, Aarhus University, 8000 Aarhus C, Denmark; Department of Microgravity and Translational Regenerative Medicine, Otto-von-Guericke-University, 39106 Magdeburg, Germany; Research Group “Magdeburger Arbeitsgemeinschaft für Forschung unter Raumfahrt-und Schwerelosigkeitsbedingungen” (MARS), Otto-von-Guericke-University, 39106 Magdeburg, Germany

**Keywords:** microgravity, tumor spheroids, cancer, cancer stem cells, space biotechnology, in vitro metastasis

## Abstract

The still young and developing space age, characterized by lunar and Martian exploration and the vision of extraterrestrial settlements, presents a unique environment to study the impact of microgravity (µ*g*) on human physiology and disease development. Cancer research is currently a key focus of international space science, as µ*g* fundamentally impacts cellular processes like differentiation, adhesion, migration, proliferation, survival, cell death, or growth of cancer cells as well as the cytoskeleton and the extracellular matrix (ECM). By creating three-dimensional (3D) tumor models in a µ*g*-environment, like multicellular spheroids (MCS), researchers can expedite drug discovery and development, reducing the need for animal testing.

This concise review analyses the latest knowledge on the influence of µ*g* on cancer cells and MCS formation. We will focus on cells from brain tumors, lung, breast, thyroid, prostate, gastrointestinal, and skin cancer exposed to real (r-) and simulated (s-) µ*g*-conditions.

## Graphical Abstract

**Figure:**
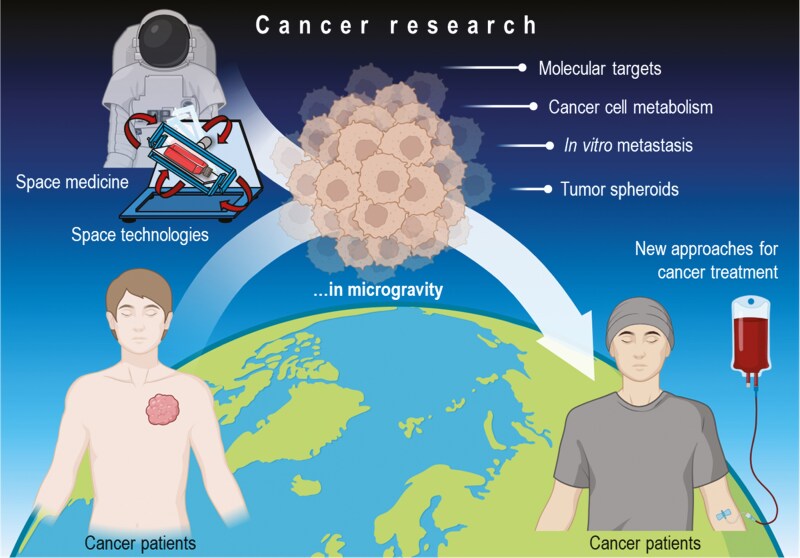
Cancer research in space is important for cancer patients on Earth. Space medicine fights cancer with the help of space technologies and microgravity research. The tumor spheroid model has a high potential for preclinical testing and development of cancer drugs and is suitable for studying cancer metabolism, progression and metastasis at the molecular level.

**Significance statement**

R-μ*g* and s-μ*g* provide unique cell culture conditions and have demonstrated cellular and molecular changes that cannot be achieved under normal gravity on Earth. Space research is becoming increasingly important for tissue engineering, translational regenerative medicine, and cancer research, where the microgravity environment induces various changes in vitro and generates multicellular spheroids and organoids that have high potential for preclinical drug testing, anticancer drug development, and for studying the processes of cancer progression and metastasis at the molecular level. This review summarizes the current state of knowledge on the effects of microgravity on cancer cells and the development of tumor spheroids.

## Introduction

Cancer is the leading cause of death, with high morbidity and mortality.^1^ About 20 million new cancer cases alongside 9.7 million deaths from cancer were measured by the Global Cancer Observatory (GLOBOCAN) in 2022.^1^ The disease is characterized by uncontrollable cell growth, spreading, and metastasis. Therefore, new research strategies to treat cancer are necessary. An innovative, albeit unconventional, approach to cancer research is the use of microgravity (µ*g*) and technology from the space sciences. This “final frontier” strategy can help to discover new targets and develop future cancer therapies.^2^

This concise review focuses on recent studies published since 2/2020, investigating the influence of µ*g* on cancer cells and the µ*g*-induced development of multicellular spheroids (MCS). MCS imitate mini-metastases and areas of solid in vivo tumors.^3^ Spheroids resemble the in vivo tumor situation better than monolayer and suspension cell cultures, thus representing a more complex model for various applications in cancer research. Therefore, MCS are imperative tools for cell biology and physiology, immunology, molecular biology, toxicology, and pharmacology. Advanced µ*g*-technology enables the engineering of scaffold-free and scaffold-containing 3D tissues and organoids.

Currently, numerous publications reporting the findings of recent space research have been published. On October 10, 2024, the PUBMED search “*cancer and spaceflight*” resulted in 272 hits and “*cancer cells and spaceflight*” listed 57 publications published since 2020, which is a strong indicator of the growing interest and research activity in the field of space medicine and cancer research in space.

This comprehensive review will summarize the current knowledge about the influence of r-μ*g* and s-μ*g* on cancer cells and MCS as metastasis models of various tumor types.

## Cancer space research

**Figure 1.**
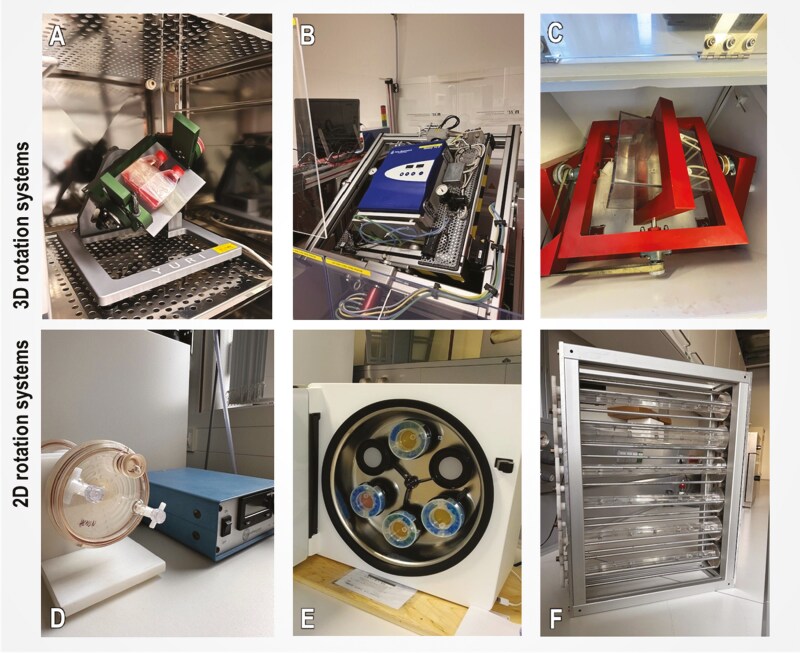
Ground based microgravity simulators. (A) Desktop random positioning machine (RPM) 2.0 by Yuri. (B) Incubator random positioning machine (iRPM) contains miniaturized CO_2_ incubator mounted on the inner rotating gimbal (developed by Prof. Jörg Sekler, University of Applied Sciences and Arts Northwestern Switzerland and the “Eidgenössische Technische Hochschule” (ETH) Zurich, Switzerland). (C) 3D clinostat designed by the Institute of Aerospace Medicine of The German Aerospace Center (DLR), Cologne, Germany. (D) Rotating wall vessel (RWV) by Synthecon Inc., Houston, TX, USA. (E) ClinoStar by CelVivo ApS, Odense, Denmark. (F) Fast rotating 2D clinostat for slide flasks developed by the Institute of Aerospace Medicine of The German Aerospace Center (DLR), Cologne, Germany.

Space research can be conducted on actual microgravity platforms, including manned spacecraft, unmanned satellites, the International Space Station (ISS), the Chinese Space Station (CSS) Tiangong, sounding rockets, parabolic flights, and drop towers.^2,4^ While real space missions are infrequent and expensive adventures, ground-based facilities like the random positioning machine (RPM), two-dimensional (2D) and 3D clinostats, and the rotating wall vessel (RWV) all offer suitable opportunities to create µ*g*-like but radiation-free conditions^4^ in our terrestrial laboratories on Earth (Figure 1). These instruments, known as ground-based facilities (GBFs), have already been presented and reviewed in detail.^2,4-7^ The simulators use different physical methods to prevent the sedimentation of cells (aggregates). In particular, the rotation-based methods cause mechanical forces on the cells that do not occur in real (space) µ*g* (r-µ*g*). If the effects on the biological samples are still comparable to those of r-µ*g*, this is referred to as “simulated µ*g”* (s-µ*g*).

Cell studies during r-µ*g* or s-µ*g* indicate that altered gravity induces stress in mammalian cells, which activates a complex adaptive response. It is widely recognized that culturing cells under conditions of r- and s-µ*g* can result in diverse changes in the genome, transcriptome, epigenome, proteome, secretome, or metabolome of both malignant and benign cells.^6,7^ Biophysically induced changes in the cytoskeleton and metabolism as well as various other biological processes often lead to reduced migration and proliferation, downregulation of MMPs and EMT-related genes by µ*g*.^8-11^ In cancer cells, some of these changes can be seen as an orientation towards a less-aggressive phenotype.^11^ However, these results vary from cell line to cell line and these changes cannot be generalized for all cancer cell lines. Most striking, however, are the cross-cancer changes in growth behavior and the development of 3D spheroids under µ*g* conditions.

Cells studied under s-μ*g* (RPM, RWV, rotary cell culture system (RCCS), or clinostats can experience shear forces, residual accelerations depending on their distance to the center of rotation, and a constant mixture of the cell culture medium. These effects may eventually lead to deviations between results from r- and s-μ*g* experiments, which makes it mandatory to confirm the results obtained from µ*g*-simulators in space. Finally, science in space and on Earth supports the development of new patient-specific treatments and brings new ideas to the fields of regenerative medicine and cancer research.

## Microgravity-induced formation of tumor spheroids

**Figure 2.**
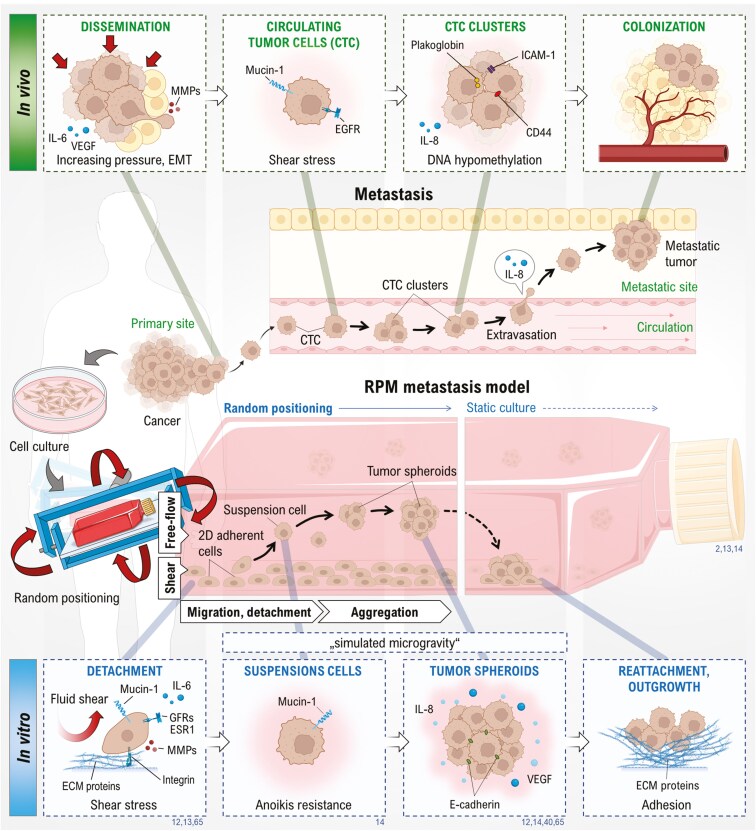
Overview of the RPM in vitro metastasis model. The RPM offers the advantage that 2D cell cultures in normal cell culture flasks can be used under conditions of simulated microgravity. The detachment of the cells and the subsequent microgravity-based spheroid formation (blue boxes) have many parallels to metastasis in the human body (green box), providing a good study model for the physiology of cancer spread and pharmaceutical tests. CD: cluster of differentiation, CTC: circulating tumor cells, GFR: growth factor receptor; ECM: extracellular matrix, EGFR: epithelial growth factor receptor; ESR1: estrogen-receptor 1, ICAM: Intercellular adhesion molecule, IL: interleukin, MMP: matrix metalloproteinase, VEGF: vascular endothelial growth factor. Parts of the figure were drawn using pictures from Biorender.com and Servier Medical Art.

The principle of spheroid formation in all GBFs (Figure 1) is based on the rotation-prevented sedimentation of suspended adherent cells. Due to their anoikis resistance, this works particularly well with cancer cells. It is now known that the successful formation of stable 3D aggregates depends on the expression of cell-cell (cadherins) and cell-ECM binding proteins (integrins) as well as anti-adhesion proteins (mucins).^12^ The RPM is the only GBF offering the advantage of using 2D cell cultures in an s-µ*g* experiment. The spheroid formation on the RPM takes place in two steps.^13^ Some of the 2D growing cells detach after some time due to the shear forces caused by the rotational movement of the RPM. This process has biological parallels to metastasis and is therefore also used as an in vitro metastasis model (Figure 2).^2,14^ Subsequently, the resuspended cells are prevented from sedimenting by the RPM movement.^13^ Over time, the floating cells grow into MCS, which are given stability by cadherins^12^ and already have structural similarities to micro-metastases. After stopping the RPM, the tumor spheroids sediment and can grow out again at the bottom of the cell culture flask. Tumor spheroids generated on the RPM show remarkably rapid and long-lasting growth and tend to have less necrosis in their center,^3,15^ as well as the formation of glandular spheroids with polarized cells in breast cancer cells.^16,17^

## Cancer stem cells in microgravity-shaped tumor spheroids

Tumor spheroids are unique because they are purposed for the enrichment of cancer stem cells (CSCs) or cells with stem cell-related characteristics,^18,19^ although this process has been little studied in µ*g*. Understanding CSCs is crucial for developing effective cancer treatments.^4,20^ Targeting CSCs can potentially eradicate the root cause of tumors and prevent disease recurrence. However, CSCs also pose significant challenges in cancer therapy due to their heterogeneity within tumors,^21^ resistance to conventional treatments, and ability to regenerate the tumor microenvironment.^22-26^ Masini et al reported that long-term exposure of pancreatic ductal adenocarcinoma cells to the RPM enhanced the expression of stem cell-like markers, which may indicate the development of a more aggressive and metastatic phenotype.^27^ However, it remains unclear from this study whether only the MCS or all cells of the RPM samples were analyzed. Mechanical stress always alters the metabolism and behaviors of cancer cells and can cause cancer cells to attain cancer stem-like cell properties, thus driving tumor progression and promoting metastasis.^28^ It is also currently unknown whether CSCs have an important influence on the development and properties of tumor spheroids formed in microgravity. This will be a field of research in the coming years. Embryonic and mesenchymal stem cells are known to react very sensitively to gravitational changes.^29-31^

## Latest studies on cancer cells exposed to microgravity conditions

### Brain tumors

A brain tumor is the abnormal growth of cells in or near the brain, including areas like the pituitary gland, nerves, pineal gland, or surrounding membranes. Tumors can also form when cancer from other parts of the body spreads to the brain. There are approximately 130 types of brain and central nervous system (CNS) tumors, ranging from rare to common. While much research has focused on cancer cells and cancer stem cells, few studies have explored how microgravity affects them.^6^

One recent example by Shaka et al aimed to explore whether r-µ*g* triggers abnormalities in cytokinesis-induced cell proliferation by using induced pluripotent human neural stem cells (NSCs) known as CS83iCTR-33n21.^32^ Using time-lapse microscopy, higher rates of abnormal cell division (ACD) in NSCs exposed to µ*g* compared to 1*g* were observed. Abnormalities included incomplete cell division (ICD) and multi-daughter cell division (MDCD), with an increased occurrence of more than two daughter cells after a single division.^32^ The findings also reveal an increased occurrence of more than two daughter cells, characteristic of a single-cell division post-cytokinesis.

The authors also investigated whether changes in NSCs during spaceflight were due to secreted molecules under µ*g*. The secretome showed similar rates of ICD, suggesting secreted molecules rather than mechanical forces drive these effects.^32^ Naïve NSCs exposed to the space-produced secretome also presented increased ICD and MDCD compared to controls. This study highlights the potential health risks of µ*g* on astronauts and may inform precautionary or therapeutic measures for long-term space exposure, though further research on the secretome’s composition is needed.^6^

### Lung cancer

Despite its significance, publications on µ*g*’s impact on lung cancer cells have been limited since 2020. All studies presented since then were performed under s-µ*g* on ground facilities; none was conducted in space or during free fall or parabolic flight maneuvers. After exposure of A549 epithelial-like lung carcinoma cells to s-µ*g*, the authors observed upregulation of *FCGBP*, *CFB*, *F5*, and *BPIFB*. According to clinical data, high gene expression of *NOX1*, *GDA* and *BPIFA1* is associated with poor outcome, while upregulation of *FCGBP* is correlated with better prognosis,^33^ which raises the question whether s-µ*g* leads to a more benign or malignant phenotype in A549 cells. Furthermore, proliferation of A549 cells decreased after 48 and 72 hours under s-µ*g*, and their morphology changed towards a granular, clumped shape. Additionally, a mesenchymal-epithelial transition was detected (significant increase in E-cadherin, decrease in N-cadherin and MMP2 expression).

In another study, A549 cells were cultivated on an RPM for 24, 36, and 48 hours. The A549 cells showed decreased proliferation with an increased number of cells in the G1 and G2 phases.^34^ S-µ*g* induced the formation of polynucleated cells and altered mitochondrial morphology, as observed via electron microscopy, with dilated cristae showing increased electron density and the presence of autophagic vacuoles. Changes in miRNA expression were detected, particularly in those regulating the cell cycle, apoptosis, and stress. Bioinformatics analyses identified that relatively few genes were responsible for these alterations in the lung cancer cells, primarily genes within the phosphatidylinositol 3-kinase-Akt (PIK3R1-Akt) signaling pathway, including the negative regulator *PTEN* and the downstream effector *FOXO3*, along with cell cycle control genes (*CDKN2A/1A* and *RB*) and genes regulating mitochondrial activity (*BCL2*), all under the influence of *TP53*.

Barkia et al recently demonstrated that different lung cancer cell lines differ in the stability of the spheroids formed on the RPM.^12^ E-cadherin and the anti-adhesive mucin-1 play important roles in this effect. The study also showed that the RPM increases the estrogen sensitivity of Calu-3 lung adenocarcinoma cells, and thus estrogens and phenol red can influence spheroid formation and stability.

### Breast cancer

Recent studies have shown that µ*g* significantly impacts breast cancer cells, affecting cytoskeletal structure, gene expression, extracellular vesicle (EV) dynamics, and spheroid formation. The following section compiles key findings, focusing on breast cancer cell lines MCF-7, MDA-MB-231, and CRL2351 under s-µ*g*.

The effect of s-µ*g* on MCF-7-derived MCS has been studied after 72 hours of RPM exposure.^35^ There were significant disruptions in cytoskeletal structure, with short, fragmented actin filaments localized around the perinuclear region and a disorganized tubulin network. These changes were associated with a marked reduction in vinculin, a focal adhesion protein, and diminished extracellular signal-regulated kinase (ERK) activation, essential for cell survival. The study suggested that disrupted focal adhesion kinase (FAK)–integrin complexes may impair ERK signaling in MCS cells under s-µ*g*, while β_1_-integrin was nearly absent in RPM-induced MCS, resulting in increased apoptosis rates.

Several studies focused on the gene expression changes in breast cancer cells and spheroids under s-µ*g*. Calvaruso et al analyzed MDA-MB-231-derived MCS after 72 h in s-µ*g* and reported upregulation of genes related to proliferation (*AKT*, *KI67*), cancer stemness (*CD44*), and metastasis (*MMP9*).^36^ Interestingly, they observed an increase in *BCL2* (an anti-apoptotic gene) and a decrease in *BAX* (a pro-apoptotic gene), suggesting a potential shift towards enhanced survival and stemness under µ*g* conditions. A related study investigated the gene expression in MCS derived from CRL2351 human breast cancer cells after five days in s-µ*g*.^37^ Upregulation of several key genes, including *BRCA1*, *RHOA*, *VIM*, *HER2*, and *MAPK1*, which are associated with cell proliferation, migration, and cytoskeletal dynamics, was observed. Notably, *HER2* was upregulated, while *RAB27A*, a gene involved in vesicle trafficking, was downregulated. These findings underscore the complexity of gene regulation under µ*g*, with potential implications for cancer progression and metastasis.

EVs have emerged as important mediators of intercellular communication and are particularly relevant in cancer biology. Thus, MDA-MB-231 cells exposed to µ*g* for 96 h showed upregulation of several Ras-like small GTPases, including RalA, RalB, RhoA, RhoC, CDC42, and Rab13, in their derived EVs.^38^ Additionally, the EV release rate decreased, but the average EV size increased, suggesting that µ*g* may alter EV biogenesis and content. Wise *et al.* extended these findings by studying MCF-7 breast cancer cells under s-µ*g* for 5 and 10 days.^39^ They observed increased secretion of EVs and higher expression levels of the tetraspanins CD81, CD63, and CD9, which are markers of exosomes. This indicates that prolonged exposure to µ*g* might enhance EV-mediated communication, potentially affecting cancer cell behavior and microenvironmental interactions.

Recent studies have examined the formation of MCS and their metastatic potential under µ*g*. MCF-7 and MDA-MB-231 cells exposed to an RPM for 14 days showed differential regulation of key mRNAs compared to 2D cultures.^40^ These findings highlighted the involvement of mRNAs related to *ERK1*, *AKT1*, *MAPK14*, *EGFR*, *CTNNA1*, *CTNNB1*, *ITGB1*, *COL4A5*, *ACTB*, and *TUBB*, as well as EGF/MAP kinase signaling pathways, with variations by cell type. Bioinformatics analyses suggested a positive association between these spheroids and the metastatic microtumor environment, indicating that µ*g* may enhance metastatic traits in breast cancer cells.

These studies reveal how µ*g* affects the breast cancer cell behavior, potentially enhancing metastasis and altering cell communication. The findings highlight the importance of µ*g*-research in cancer biology and its potential for developing new therapies, especially for aggressive metastatic breast cancers.

### Thyroid cancer

The most studied thyroid cancer cell line under spaceflight conditions in recent years is the follicular thyroid cancer (FTC)-133 cell line. As part of the CellBox-1 experiment, FTC-133 cells were cultured on the ISS for 12 days.^41,42^ Differences between flight and ground supernatant samples were found in the number of secreted exosomes and the distribution of tetraspanins on their surface.^42^ Exosomes are known to regulate the surrounding microenvironment of tumors and can modulate immune and therapy responses, and promote angiogenesis, tumorigenesis, and ECM remodeling.^43-47^ Tetraspanins are involved in cancer cell motility, metastasis, tumor initiation, progression, and angiogenesis.^48,49^ Particularly, a higher content of CD63 and CD81 tetraspanins was found in flight samples. CD63 and CD81 expression are associated with tumor invasion and tumor growth, respectively.^50,51^ Additionally, a total of 119 miRNAs were altered, of which 23 were associated with thyroid cancer.^41^ These included the downregulation of hsa-miR-429, hsa-miR-199, and hsa-miR-128-3p, which have been described as tumor suppressors.^52-54^

The CellBox-2 experiment reported the formation of MCS from FTC-133 cells cultured for 5 and 10 days on the ISS. Changes in gene expression included ECM components (*COL1A1, ITGB1*), cell adhesion factors (*CAV1, ICAM1, SRC, VCL, PXN*), growth factors (*EGFR*), cytokines (*IL6, CXCL8*), and other signaling molecules (*RELA, ERK1, ERK2*).^55^ MCS formation from thyroid cancer cells under s-µ*g* has been reported recently for multiple cell lines, including FTC-133, WRO, and the Korean SNU-790 and SNU-80.^14,56^ The transition from 2D to 3D growth is considered a more physiologically relevant tumor model and allows for the study of drugs and possible therapy targets.^57,58^ Dexamethasone, a synthetic glucocorticoid used in combination with cancer therapeutics, selectively inhibits MCS formation in a dose-dependent manner in FTC-133 cells exposed to s-µ*g*.^14,57,59,60^ Based on the analysis, components of the ECM (laminin, fibronectin, MMP-9), anti-adhesion molecules (mucin-1), cell junction (E-cadherin, β-catenin, FAK), p38, and the phosphorylated glucocorticoid receptor are possible targets of dexamethasone.^14^ Finally, it was recently reported that cell detachment and MCS formation in FTC-133 cells are independent processes affected by different conditions such as cell flask geometry, averaged gravity vector direction over time, angular velocity, fluid flow, and amount of air bubbles.^13^

### Prostate cancer

Prostate cancer cells exposed to μ*g* conditions offer a unique opportunity to advance our understanding of cancer mechanisms and identify novel therapeutic strategies, and the prostate cancer cell line PC-3 has been a much-valued model for studies under r- and s-μ*g* conditions. Schulz et al took advantage of a parabolic flight opportunity to investigate early responders across the whole transcriptome to altered µ*g* conditions.^61^ Adaptations of the F-actin cytoskeleton-like stress fibers and pseudopodia were visible after the first parabola. In addition to an identified network of relevant prostate cancer cytokines and chemokines differentially expressed under µ*g* (*IL6 and CXCL8*), regulatory lncRNAs and microRNAs were found to be altered under µ*g* (*miR221/222, MIR222HG, MIR3142HG, and LINC02605*). In total, 298 potential biomarkers were identified, which were potentially relevant markers for carcinogenesis and inflammatory pathways.

It is known that PC-3 cells exposed to s-μ*g* conditions form MCS. PC-3 cells were grown on the RPM for 3 and 5 days.^62^ The vascular endothelial growth factor (VEGF) signaling components (*VEGFA, FLK1, FLT1, SRC1, AKT1, MTOR, ERK1/2, LCN2*) were found to be altered in MCS compared to 1*g* controls as well as components of the cytoskeleton (*ACTB, TUBB*), ECM (*LAMA3, COL1A1, FN1, COL4A5*), and focal adhesion complex (*VCL, CDH1*). Since most of these components were upregulated, the authors suggested that µ*g* may affect the regulation of tumor progression and angiogenesis.^62-64^ Dietrichs et al cultured PC-3 cells on the RPM for up to 24 hours to study the early cytokine gene expression and the secretion profile.^65^ After 24 hours, MCS formation was detected and an increased cytokine gene expression of *IL6*, *CXCL8*, and *IL1A* was reported. Cytoskeleton components (*ACTB, MSN*) were also increased in MCS after 24 hours along with ECM components (*FN1, COL1A1, TIMP1,* and *LAMA3)*. The PAM, MAPK, and VEGF signaling pathways were proposed as the underlying pathways causing MCS formation in PC-3 cells, for which an increased gene expression of *VEGF, FLT1,* and *EGFR* was reported.^65^ All in all, PC-3 cells exposed to s-μ*g* demonstrate MCS formation, a proinflammatory phenotype, as well as alterations of the cytoskeleton, ECM, and focal adhesion complex when indicating their impact on the growth and progression of these cells.

### Gastrointestinal tumors

In recent years, no specific emphasis was directed on µ*g*-induced spheroid formation in gastrointestinal or liver tumors, only a few studies have focused on the influence of the µ*g*-environment on the metabolism and chemoresistance of gastrointestinal cancer cells. As described by Chen et al, an RCCS had a major effect on the lipid metabolism of highly malignant HGC-27 gastric cancer cells and induced glycolytic pathways.^66^ Rembiałkowska et al described that µ*g* attenuates the effect of genes associated with drug resistance in gastric cancer cells, which could be of benefit in case of resistance to chemotherapy. In their likewise RCCS-based study, s-µ*g* altered the expression of multidrug resistance genes in EPG85-257 cells, induced changes in cancer cell survival and in the cellular cytoskeleton, making the cells more susceptible to chemotherapy.^67^ Masini et al found that pancreatic cancer cells cultured on an RPM were transformed toward a more stem and aggressive phenotype. The observed restructuring of actin, formation of tumor spheroids, and enhancement of epithelial-to-mesenchymal transition may also affect the susceptibility of the tumor cells to drugs and lead to the acquisition of an aggressive and metastatic stem cell-like phenotype.^27^

Smit et al aimed to create a new 3D model for pharmaceutical tests by culturing encapsulated LS180 colorectal cancer spheroids on a clinostat. After 20 days of s-µ*g* culture and subsequent treatment with paclitaxel, the spheroid model proved to be suitable for testing new chemotherapeutic agents against colorectal cancer.^68^

### Skin cancers (melanoma)

Since 2020, only one original study looking at the effects of s-µ*g* on skin cancers could be identified. This study used a “Lab-on-a-Chip” (LOC) device.^69^ Although some inhibiting influence of the LOC environment on cell viability was partially observed, viability increased for HaCaT and A375 cells under s-µ*g* by clinorotation compared to 1*g*. Mitochondrial activity increased under s-µ*g* but was again partially inhibited by the LOC environment. Proliferation of HaCaT and A375 cells was inhibited by s-µ*g* and furthermore, cell morphology changed towards a shrunken and round shape. The cells developed lamellipodia, decreased filopodia and stress fibers, and actin fibers were disrupted and arranged in peripheral parts of the cytoplasm in both cell lines. Going forward, a LOC may help to facilitate research under r- and s-µ*g* conditions by providing initial analysis tools right within the µ*g* environment, enabling more direct and more “in-time” data generation.

## The spheroid model as new option for pharmacological research

Cancer cell spheroids, which are 3D clusters of cancer cells, are increasingly utilized in pharmacological research because they closely mimic the tumor microenvironment (TME) found in the human body.^70^ Various methods for generating spheroids include the hanging drop method,^71^ liquid overlay method, spinner flask method, microfluidic devices, magnetic levitation, and the scaffold method, each with unique advantages and limitations in modeling tumor biology. Spheroids are essential for studying tumor characteristics, such as cell morphology, gene expression, and interactions with the surrounding environment, including immune cells.^72^ Tumor-associated macrophages, prevalent in the TME of solid tumors and linked to poor patient outcomes, can be incorporated into spheroids to investigate cancer-immune cell interactions crucial for developing immunotherapies.^70,73^

One of the unique characteristics of spheroids is their ability to become hypoxic at the core, a trait shared with many solid tumors. This unique characteristic makes spheroids an ideal platform for studying how cancer cells adapt to low-oxygen conditions, a crucial aspect of cancer development. The potential of spheroids in this area is intriguing and inspiring, as it opens new possibilities for testing drugs that target hypoxic cells and understanding the adaptive mechanisms of cancer cells.^70,72,73^ In a recent study, endothelial cell culture on porous microfluidic channel surfaces was magnetically attached to tumor spheroids generated on a composite polymer-hydrogel microwell plate, which allowed the study of the pro-angiogenic endothelial cell organization and prevented the angiogenesis by inhibition of the VEGF pathway and the Notch signaling pathway.^74^

Due to the replication of the complex cell-cell-matrix interactions, nutrients, and oxygen gradients found in actual tumors, spheroids are a more accurate model for testing the effectiveness of anticancer drugs than traditional 2D cell cultures. However, the lack of generation of spheroids with uniform size and shape limits their usage in anti-cancer research and high-throughput drug screening. Even though the hanging drop method and liquid overlay technique allow for the generation of uniform spheroids, which can be used in high-throughput drug screening^75,76^ they are labor-intensive for large-scale production,^77^ resulting in low-throughput fabrication and variation in the spheroid sizes. However, combining the spheroid production with culture in microfluidic devices allows controllable cell perfusion, simulation of fluid shear effects, and mimicking of the microenvironment-like in vivo conditions improved the approach for drug testing.^78,79^

In summary, 3D tumor spheroids are valuable for screening anti-cancer drugs, assessing cytotoxicity, investigating tumor cell behavior, evaluating treatment resistance and immunotherapy effects, providing insights that enhance the effectiveness of targeted cancer therapies.^80,81^

## Conclusion and future perspectives

The age of space exploration has just begun. Private companies offer short-term trips into space. Moon and Mars missions are planned soon. Since 2020, many publications have appeared reporting data from space research. On October 10, 2024, a PubMed search for “microgravity” and “cells” since 2020 gave 621 hits and for “spaceflight” and “cells” 449 results were obtained. This review discusses the recent literature about the effects of μ*g* on cancer cells and the generation of 3D tumor spheroids, as an important common finding is that exposure of a variety of cancer cells to spaceflight or μ*g*-simulating devices triggers the aggregation of cancer cells in vitro. The current literature reports on spheroid and organoid formation (Table 1) which might be used as metastasis and neoangiogenesis models for different cancer-type therapies.^2^

**Table 1.** Summary of review articles addressing cancer cells exposed to microgravity conditions and 3D MCS formation.

Cell line	Origin	Microgravity platform	Timeline	Findings	Ref.
Brain tumors					
CS83iCTR-33n21	Neural stem cells	ISS	39,6 d	Incomplete cell division; impaired cytokinesis; multi-daughter cell division and alterations in the secretome	^32^
Lung cancer					
A549	Lung carcinoma	RWV/ hanging drop	48, 72 d	Reverted epithelial-to-mesenchymal transition phenotype; impaired proliferation and cell morphological changes	^33^
		RPM	24, 36, 48 h	Impaired proliferation; presence of polynucleated cells; altered morphology of mitochondria and alterations in miRNAs involved in cell cycle regulation, apoptosis, and stress	^34^
Calu-3	Lung adenocarcinoma		3 d	Increased estrogen sensitivity and altered spheroid stability	^12^
NCI-H1703	Lung squamosal carcinoma			Altered spheroid stability	
MCF-7	Breast carcinoma		72 h	Disruption of actin and tubulin network in MCS; reductions in Vinculin; ERK inactivation and observed apoptosis	^35^
Breast cancer					
MDA-MB-231	Breast carcinoma	RPM	72 h	Upregulation of genes related to proliferation, cancer stemness and metastasis	^36^
CRL2351	Breast adenocarcinoma		5 d	Gene analysis Upregulation of genes related to proliferation, migration and cytoskeletal dynamics Downregulation of genes involved in vesicle trafficking Cytoskeletal rearrangement into a spherical shape	^37^
MDA-MB-231	Breast carcinoma	Gravite system	96 h	Upregulation of Ras-like small GTPases; allterations in extracellular vesicles biogenesis and content	^38^
MCF-7	Breast carcinoma	RPM	5, 10 d	Increased secretion of extracellular vesicles; increased exosome marker expression such as CD81, CD63, and CD9	^39^
MDA-MB-231	Breast carcinoma		14 d	Involvement of novel genes related to cellular signaling pathways (MAPK, PAM, and VEGF) in spheroids	^40^
Thyroid cancer					
FTC-133	Follicular thyroid carcinoma	ISS	12 d	Altered exosome subpopulation	^42^
			5, 10 d	Changes in gene expression of ECM components, cell adhesion factors, growth factors, cytokines and others such as *RELA, ERK1, ERK2*	^55^
		RPM	4 h, 3 d	Dexamethasone inhibits MCS formation; possible targets include ECM components, anti-adhesion molecules, cell junctions and phosphorylated glucocorticoid receptor	^14,57^
			1, 3 d	MCS formation influenced by cell flask geometry, averaged gravity vector direction, angular velocity, fluid flow, and air bubbles	^13^
Prostate cancer					
PC-3	Prostate adeno-carcinoma	Parabolic flight	/	Presence of F-actin stress fibers and pseudopodia; early differential expression of IL6 and CXCL8; alterations in regulatory lncRNAs and microRNAs	^61^
		RPM	3, 5 d	Alterations in VEGF signaling components, cytoskeleton components, ECM components, and the focal adhesion complex, suggest a more aggressive cancer phenotype under microgravity conditions	^62^
			0.5, 2, 4 and 24 h	Gene analysis Upregulation of genes related to cytokine production such as *IL6*, *CXCL8*, and *IL1A* Upregulation of genes related to cytoskeleton components and ECM components Activated PAM signaling pathways in MCS	^65^
Gastrointestinal cancer					
HGC-27	Gastric carcinoma	RCCS	1,3 d	Induced glycolytic pathways	^66^
EPG85-257	Gastric carcinoma		24, 48, 72, 96 h	Altered expression of multidrug resistance genes; cytoskeletal reorganization; changes in cell survival; increased cell susceptibility to chemotherapy	^67^
PaCa-44	Pancreatic adenocarcinoma	RPM	1, 7, 9 d	Presence of an aggressive and metastatic stem cell-like phenotype; actin reorganization, enhanced epithelial-to-mesenchymal transition	^27^
CFPAC-1	Pancreatic adenocarcinoma				
LS180	Colorectal adenocarcinoma	Clinostat	20 d	Verified spheroids as a suitable model for pharmaceutical testing	^68^
Skin cancer					
HaCaT	Aneuploid immortal keratino-cyte	Clinostat	2 h	Increased viability and mitochondrial activity; inhibited proliferation; altered cell morphology with a shrinked and rounded shape and lamellipodia but decreased filopodia and stress fibers; showed a disrupted actin network	^69^
A375	malignant melanoma				

In vitro investigations with focus on the mechanisms for spheroid generation using OMICS technology and in silico methods can enhance the current knowledge about in vivo epithelial to mesenchymal transition, cancer progression, and metastasis. Furthermore, this new approach technology referring to biomedical space research provides new information by applying in vitro (human cancer cell culture), in silico/artificial intelligence or in chemico (ie, pharmacological/toxicological tests) technologies. By studying cancer cells in microgravity, researchers can observe how tumors grow and respond to treatments in a way that better mimics the behavior of tumors in the human body than conventional 2D culture models. These findings could pave the way for improved drug screening platforms that test treatments under more realistic, 3D conditions, leading to more effective therapies tailored to the genetic and molecular profiles of individual patients. In this way, space medicine is helping to develop novel therapies that can be translated to in vivo models while reducing the use of laboratory animals.

Although real space conditions still leave more questions for cancer progression, including the inclusion of space radiation and a compromised immune system under space conditions, the knowledge gained from space research is already supporting medicine and research on Earth.

## Data Availability

Data sharing is not applicable to this article as no new data were created or analyzed in this study.
